# Sphingosine-1-Phosphate Facilitates Skin Wound Healing by Increasing Angiogenesis and Inflammatory Cell Recruitment with Less Scar Formation

**DOI:** 10.3390/ijms20143381

**Published:** 2019-07-10

**Authors:** Masayo Aoki, Hiroaki Aoki, Partha Mukhopadhyay, Takuya Tsuge, Hirofumi Yamamoto, Noriko M. Matsumoto, Eri Toyohara, Yuri Okubo, Rei Ogawa, Kazuaki Takabe

**Affiliations:** 1 Department of Plastic, Reconstructive and Aesthetic Surgery, Nippon Medical School, Tokyo 113-8603, Japan; 2 Division of Surgical Oncology, Department of Surgery, Virginia Commonwealth University School of Medicine and Massey Cancer Center, Richmond, VA 23298-0011, USA; 3 Department of Surgery, The Jikei University School of Medicine, Tokyo 105-8461, Japan; 4 Department of Molecular Pathology, Osaka University, Suita 565-0871, Japan; 5 Division of Breast Surgery, Department of Surgical Oncology, Roswell Park Comprehensive Cancer Center, Buffalo, NY 14263, USA; 6 Department of Surgery, University at Buffalo Jacob School of Medicine and Biomedical Sciences, the State University of New York, Buffalo, NY 14203, USA

**Keywords:** sphingosine-1-phosphate, sphingosine kinase-1, sphingosine1-phosphate receptor-2, skin wound healing

## Abstract

Wound healing starts with the recruitment of inflammatory cells that secrete wound-related factors. This step is followed by fibroblast activation and tissue construction. Sphingosine-1-phosphate (S1P) is a lipid mediator that promotes angiogenesis, cell proliferation, and attracts immune cells. We investigated the roles of S1P in skin wound healing by altering the expression of its biogenic enzyme, sphingosine kinase-1 (SphK1). The murine excisional wound splinting model was used. Sphingosine kinase-1 (SphK1) was highly expressed in murine wounds and that SphK1^−/−^ mice exhibit delayed wound closure along with less angiogenesis and inflammatory cell recruitment. Nanoparticle-mediated topical SphK1 overexpression accelerated wound closure, which associated with increased angiogenesis, inflammatory cell recruitment, and various wound-related factors. The SphK1 overexpression also led to less scarring, and the interaction between transforming growth factor (TGF)-β1 and S1P receptor-2 (S1PR2) signaling is likely to play a key role. In summary, SphK1 play important roles to strengthen immunity, and contributes early wound healing with suppressed scarring. S1P can be a novel therapeutic molecule with anti-scarring effect in surgical, trauma, and chronic wound management.

## 1. Introduction

Wound healing is a dynamic and complex process that consists of sequential, albeit somewhat overlapping, inflammatory, proliferative, and remodeling phases [1,2,3,4]. In the inflammatory phase, immune cells (particularly macrophages) are recruited into the wound [3,5]. Inflammatory cells not only sterilize the wound; they also generate a finely balanced assortment of factors that promotes the rapid healing in the proliferative phase [3,5], which includes angiogenesis [6]. The topical wound treatments that are currently available target some of these factors (e.g., prostaglandin E1 and basic fibroblast growth factor). Recent studies have shown that fatty acids and their G protein-coupled receptors may also be important targets of novel wound healing treatments: several studies showed that the fatty acid receptors GPR40 and GPR120 play important roles in wound healing processes such as cell migration [7,8]. In addition, natural products such as honey, alkaloids, flavonoids, tannins, saponins, and polyphenols have been shown to promote wound healing [9,10]. We speculate here that additional emerging therapeutic targets in skin wound healing may be the sphingolipids and their biogenic enzymes.

Sphingosine-1-phosphate (S1P) is generated by sphingosine kinase-1 (SphK1) and -2 (SphK2), which are located in the cytosol and nucleus, respectively. Only SphK1-generated S1P is transported out of the cell [11]. It then binds in a paracrine or autocrine manner to S1P-specific G protein-coupled receptors (S1PR), of which there are five forms.

This binding event regulates various physiological processes in the S1P-binding cell [12], as follows. First, the binding of S1P to S1PR regulates lymphocyte trafficking, including the recruitment of inflammatory cells into inflamed tissues [13,14,15]. This effect is mediated by the S1P concentration gradient between various tissues: this gradient shapes the egress of S1PR1-expressing lymphocytes from secondary lymphoid organs into the blood or lymphatic vessels [16,17]. This mechanism has been targeted for the treatment of multiple sclerosis: Fingolimod (FTY720), which is a functional agonist of S1PR, induces lymphocytes to sequester in lymph nodes, thereby preventing them from contributing to the autoimmune reaction that causes the disease [12]. S1P-S1PR binding also acts to retain inflammatory cells in inflamed tissues, which produce high levels of S1P [13,18,19,20].

Second, S1P-S1PR binding plays key regulatory roles in vasculogenesis, angiogenesis, and blood vessel permeability [13,18,19,20]. Specifically, S1P regulates angiogenesis by binding to S1PR1 and S1PR3 on vascular endothelial cells, thereby inducing them to form capillary-like networks [18]. Moreover, S1P (and its functional analog FTY720) increases adherens junction assembly in endothelial cells: as a result, S1P treatment potently inhibits VEGF-induced endothelial cell transmonolayer permeability in vitro and vascular permeability in mice [21]. Since there are high levels of S1P in the blood, this vascular permeability-related activity of S1P also helps maintain the endothelial barrier integrity of specific vascular beds. This function of S1P is mediated by endothelial cell S1PR1 [22]. By contrast, S1P binding to S1PR2 disrupts endothelial barrier permeability [23]. These disparate effects of the S1PRs are due to the fact that S1PR1 couples solely with Gi/o whereas S1PR2 couples with Gq, G12, and G13 as well as Gi/o. The activation of G12 and G13 stimulates the small GTPase Rho, which induces cortical actin destabilization, stress-fiber formation, and endothelial barrier disruption [22,24].

Given that S1P promotes lymphocyte recruitment to and retention in inflamed tissues along with vasculogenesis and angiogenesis, we hypothesized that S1P is involved in the skin wound healing process by enhancing the local recruitment of the inflammatory cells that produce various wound healing-related factors in the wound. The aim of this study was to clarify the roles of the SphK1/S1P axis in the wound healing process.

## 2. Results

### 2.1. Longitudinal SphKs and S1PRs Expression during Mouse Wound Healing

**Figure 1:**
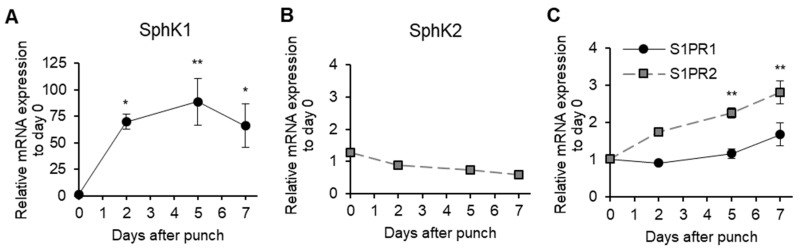
Longitudinal sphingosine-1-phosphate (S1P) production during mouse wound healing. Splinted excisional wounds (*n* = 4–6) were generated in C57BL/6J mice, and the mRNA expression of (**A**) sphingosine kinase-1 (SphK1), (**B**) sphingosine kinase -2 (SphK2), and (**C**) sphingosine-1-phosphate reseptor-1/2 (S1PR1/2) in the wound during wound healing was measured. All values shown in this figure represent the mean ± s.e.m. * *p* < 0.05, ** *p* < 0.01.

To test our hypothesis, we first investigated S1P signaling during wound healing using the murine excisional wound splinting model [25]. SphK1 expression in the wound started increasing on Day 2 and peaked on Day 5 (88.6-fold increase compared with immediately after the wounds were generated) (Figure 1A). SphK2 expression did not change (Figure 1B). Interestingly, the expression of S1PR2 (which inhibits S1PR1 and S1PR3 signaling [12]) gradually increased towards the end of the wound healing process. S1PR1 expression did not change significantly (Figure 1C). S1PR3 expression was not detected at any time point. Thus, SphK1, but not S1PR1, is massively upregulated in the proliferative phase of wound healing.

### 2.2. Effect of SphK1 Gene Knockout on Wound Healing, Vasculogenesis, and Cell Proliferation

**Figure 2:**
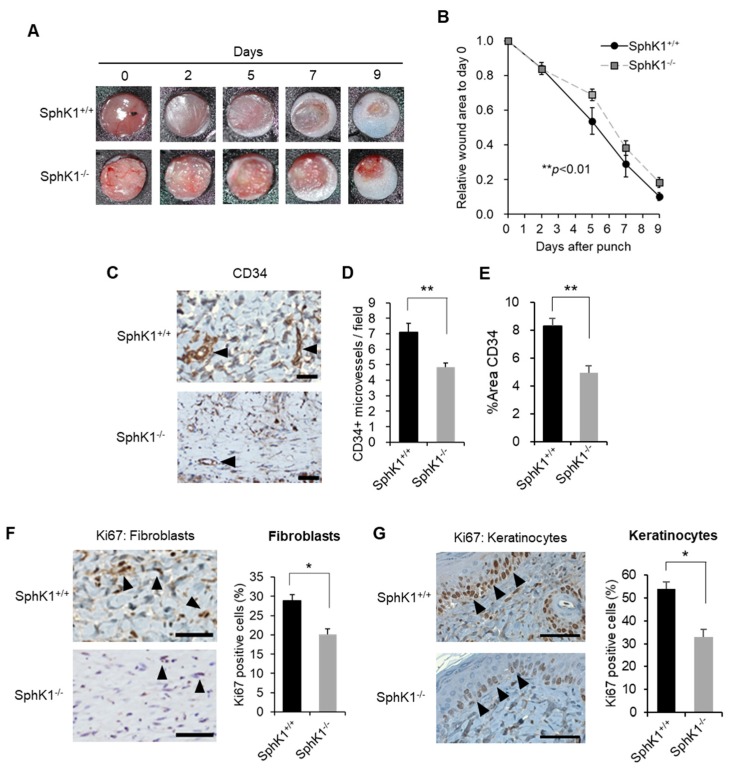
Effect of SphK1 knockout on wound healing, vasculogenesis, and cell proliferation. (**A**) Splinted excisional wounds were generated in SphK1^−/−^ and SphK1^+/+^ mice. Representative images of the closing wounds are shown. (**B**) Change in wound area over time (*n* = 6–10). (**C**) Representative images of CD34 expression on immunohistochemistry are shown. Arrowheads indicate positive findings (scale bars: 50 µm). (**D**) The numbers of CD34-positive microvessels per 200-fold magnified field are graphed (*n* = 4). (**E**) The percentage of the wound area that is occupied by CD34^+^ cells is graphed (*n* = 4). (**F**) Representative images of Ki67 expression in fibroblasts on immunohistochemistry are shown. Arrowheads indicate positive findings (scale bars: 50 µm). The frequencies of Ki67^+^ fibroblasts are graphed (*n* = 4). (**G**) Representative images of Ki67 expression in keratinocytes on immunohistochemistry are shown. Arrowheads indicate positive findings (scale bars: 50 µm). The frequencies of Ki67^+^ keratinocytes are graphed (*n* = 4). All values shown in this figure represent the mean ± s.e.m. * *p* < 0.05, ** *p* < 0.01.

Compared with littermate wild-type (WT) mice, SphK1^−/−^ mice had significantly delayed wound healing, as determined by two-factor repeated measures ANOVA (Figure 2A,B; *p* = 0.010). This reflects the tendency of the SphK1^−/−^ mice to have larger wound sizes on Days 5, 7, and 9 after injury, as determined by Student’s *t*-test (*p* = 0.056, 0.262, and 0.068, respectively). We investigated vasculogenesis on Day 5 by immunohistochemistry against CD34, which is an early marker of vasculogenesis [26]. The SphK1^−/−^ mice exhibited significantly less angiogenesis than the WT mice (Figure 2C–E). Immunohistochemistry with Ki67 showed that SphK1 knockout also had similar suppressive effects on the proliferation of both the fibroblasts in the wound (Figure 2F) and the keratinocytes at the wound edge (Figure 2G). The Ki67^+^ cells in the latter analyses are expressed as the percentage of Ki67^+^ cells/total cells per field.

### 2.3. Effect of SphK1 Gene Knockout on Inflammatory Cell Recruitment during Wound Healing

**Figure 3:**
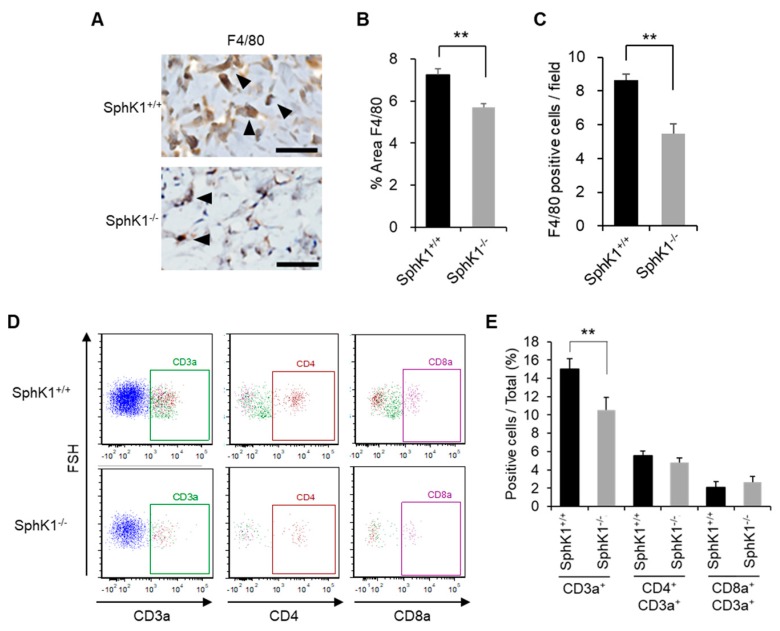
Effect of SphK1 knockout on inflammatory cell recruitment during wound healing. (**A**) Representative images of F4/80 expression on immunohistochemistry are shown. Arrowheads indicate positive findings (scale bars: 50 µm). (**B**) The percentage of the wound area that is occupied by F4/80^+^ cells is graphed (*n* = 4). (**C**) The number of F4/80^+^ cells per field is graphed (*n* = 4). (**D**,**E**) Representative flow cytometric plots (D) and frequency of the indicated T cell populations (E) on Day 5 after wounding, as determined by flow cytometry (*n* = 5–8). All values shown in this figure represent the mean ± s.e.m. ** *p* < 0.01.

Immunohistochemistry showed that the SphK1^−/−^ mice exhibited significantly decreased macrophage numbers compared to the WT mice on Day 5 (Figure 3A–C). Flow cytometric analyses confirmed that the SphK1^−/−^ mice had significantly lower frequencies of T cells in the wound five days after injury (Figure 3D,E).

### 2.4. Effect of Nanoparticle-Mediated Topical SphK1 Gene Delivery on Wound Healing, Vasculogenesis, and Cell Proliferation

**Figure 4:**
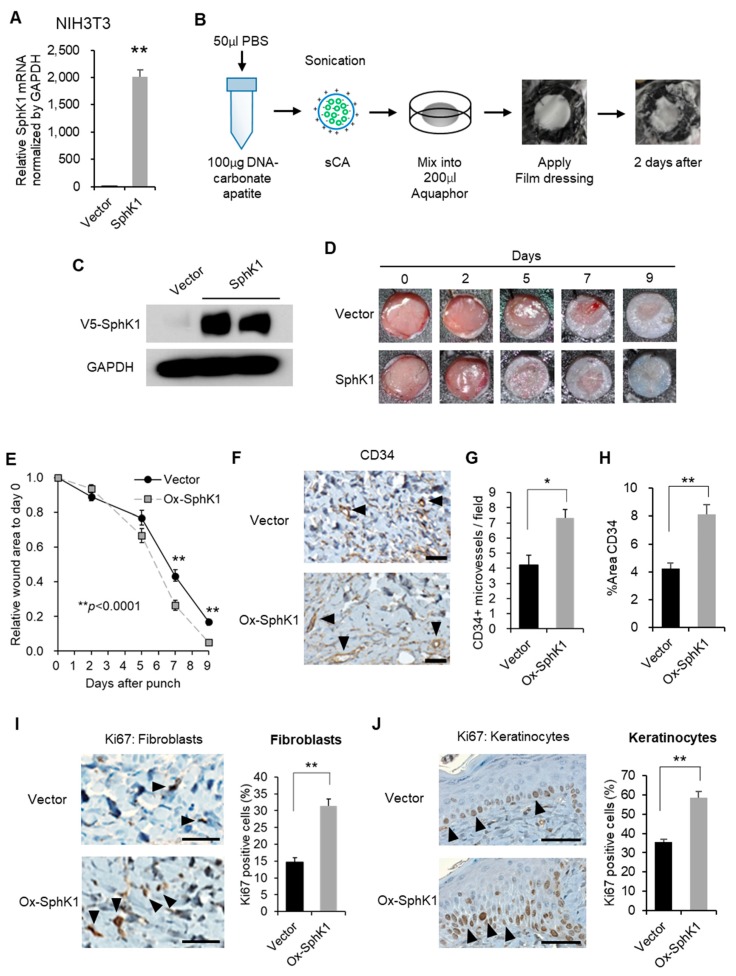
Effect of SphK1 overexpression on wound healing, vasculogenesis, and cell proliferation. (**A**) In vitro transfection efficiency with SphK1-expressing plasmid using super carbonate apatite (sCA) in NIH3T3 cells. (**B**) An ointment containing a SphK1-expressing plasmid encapsulated with sCA was prepared. (**C**) In vivo transfection efficiency of the sCA-encapsulated plasmid, as shown by immunoblots of V5-SphK1 expression in the wound surface tissues two days after application. (**D**) Representative images of the closing wounds are shown. (**E**) Effect of the plasmid ointment on wound closure. The change in wound area over time is graphed (*n* = 12). (**F**) Representative images of CD34 expression on immunohistochemistry are shown. Arrowheads indicate positive findings (scale bars: 50 µm). (**G**) The numbers of CD34-positive microvessels per 200-fold magnified field are graphed (*n* = 4). (**H**) The percentage of the wound area that is occupied by CD34^+^ cells is graphed (*n* = 4). (**I**) Representative images of Ki67 expression in fibroblasts on immunohistochemistry are shown. Arrowheads indicate positive findings (scale bars: 50 µm). The frequencies of Ki67^+^ fibroblasts are graphed (*n* = 4). (**J**) Representative images of Ki67 expression in keratinocytes on immunohistochemistry are shown. Arrowheads indicate positive findings (scale bars: 50 µm). The frequencies of Ki67^+^ keratinocytes are graphed (*n* = 4). All values shown in this figure represent the mean ± s.e.m. * *p* < 0.05, ** *p* < 0.01.

We generated control and SphK1-expressing plasmids that were encapsulated with super carbonate apatite (sCA). sCA is a nanoparticle that is safe for in vivo gene delivery. The in vitro and in vivo safety of sCA-mediated gene delivery has been reported [27]. When mixed with sCA, the control and SphK1-expressing plasmids transfected mouse dermal fibroblast NIH3T3 cell lines in vitro with high efficiency (Figure 4A). Ointments containing the sCA-encapsulated plasmids were then generated and applied topically to the wounds of wound splinting model mice (Figure 4B). V5-tag protein expression analysis showed that the plasmids had a high transfection rate in vivo (Figure 4C). Compared with the vector, the SphK1 plasmid significantly accelerated wound closure, as determined by two-factor repeated measures ANOVA (*p* < 0.0001, Figure 4D,E). This reflected significantly greater closure on Days 7 and 9 after injury (*p* = 0.003 and 0.0002, respectively), as shown by Student’s *t*-test (the vector and SphK1 plasmid did not differ significantly in terms of Day 5 closure rate; *p* = 0.122). We then investigated vasculogenesis and cell proliferation on Day 5 by immunohistochemistry. The SphK1 plasmid ointment significantly accelerated vasculogenesis (Figure 4F–H). Immunohistochemistry with Ki67 showed that the SphK1 plasmid ointment had corresponding positive effects on the proliferation of both the fibroblasts in the wound (Figure 4I) and the keratinocytes at the wound edge (Figure 4J). The Ki67^+^ cells in the latter analyses are expressed as the percentage of positive cells/total cells per field.

### 2.5. Effect of Nanoparticle-Mediated Topical SphK1 Gene Delivery on Inflammatory Cell Recruitment during Wound Healing

**Figure 5:**
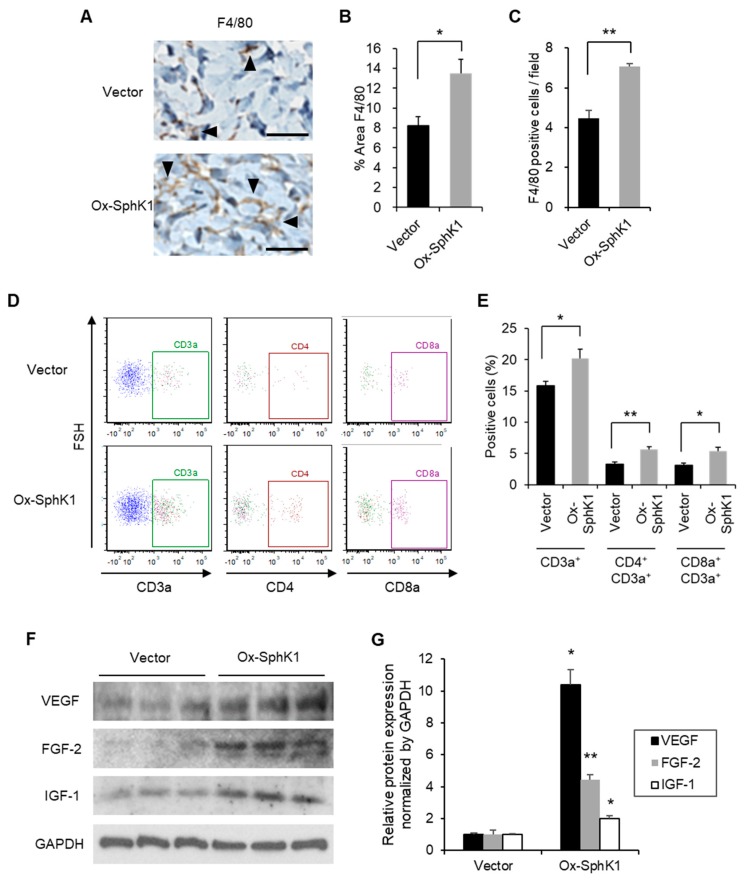
Effect of SphK1 overexpression on inflammatory cell recruitment and enhanced wound-related factors. (**A**) Representative images of F4/80 expression on immunohistochemistry are shown. Arrowheads indicate positive findings (scale bars: 50 µm). (**B**) The percentage of the wound area that is occupied by F4/80^+^ cells is graphed (*n* = 4). (**C**) The number of F4/80^+^ cells per field is graphed (*n* = 4). (**D**,**E**) Representative flow cytometric plots (D) and frequency of the indicated T cell populations (E) on Day 5 after wounding, as determined by flow cytometry (*n* = 5–8). (**F**) Effect of the plasmid ointment on the expression of the indicated wound healing-related factors on Day 5 after wounding, as determined by immunoblot analysis. (**G**) The immunoblots were quantified and the data were graphed (*n* = 3). All values shown in this figure represent the mean ± s.e.m. * *p* < 0.05, ** *p* < 0.01.

Immunohistochemistry showed that the SphK1 plasmid-treated wounds had significantly higher macrophage numbers on Day 5 (Figure 5A–C). Moreover, the SphK1 plasmid ointment increased the recruitment of total T cells, CD4 T cells, and CD8a T cells in the wound five days after injury (Figure 5D,E). It should be noted that uninjured SphK1^−/−^ mice exhibit normal lymphocyte trafficking despite the fact that their blood S1P levels are about half of those in WT mice [28]. Thus, our experiments suggest that SphK1 participates in the recruitment of inflammatory cells to the wound, and that this is needed for the normal progression of the proliferative phase of wound healing. These results are consistent with our hypothesis that after wounding, S1P generated by SphK1 promotes vasculogenesis and recruits inflammatory cells, including lymphocytes and macrophages, and that this facilitates the wound healing process during the proliferative phase. Furthermore, immunoblot analyses showed that the SphK1 plasmid ointment increased expression of the well-known wound healing-related factors VEGF, FGF-2, and IGF-1 [29,30,31] in the wound on Day 5 (Figure 5F,G). These findings suggest that these wound-related factors were secreted by the recruited lymphocytes and macrophages.

### 2.6. Effect of SphK1 Overexpression on Granuloma Formation

**Figure 6:**
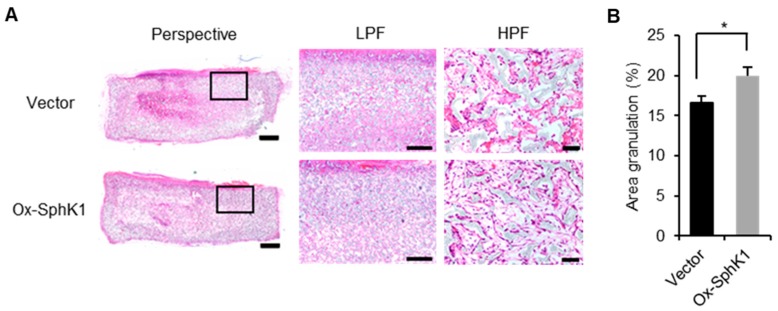
Topical SphK1 gene delivery promotes granulation. (**A**) Representative images of the hematoxylin and eosin (HE)-stained sponge granulomas treated with sCA-encapsulated plasmid injection on Day 14 are shown. The black boxes are shown magnified. (scale bars: Perspective: 1 mm; LPF: 400 µm; HPF: 50 µm). (**B**) Percentage of granulated area is graphed (*n* = 8). All values in this figure represent the mean ± s.e.m. * *p* < 0.05.

When we injected sponge granulomas in mice with the sCA-encapsulated vector or SphK1 plasmid every other day, as described previously [32], the SphK1 plasmid generated clearer collagen bundles, higher fibroblast density, and less dead cell accumulation in the center of the sponge on Day 14 (Figure 6A). Moreover, on Day 14 after injury, the SphK1 plasmid associated with significantly more granulation than the control plasmid (Figure 6B).

### 2.7. Effect of SphK1 and S1PR2 Gene Expression on Scar Thickness, the Interaction between Transforming Growth Factor (TGF)-β1 and S1P

**Figure 7:**
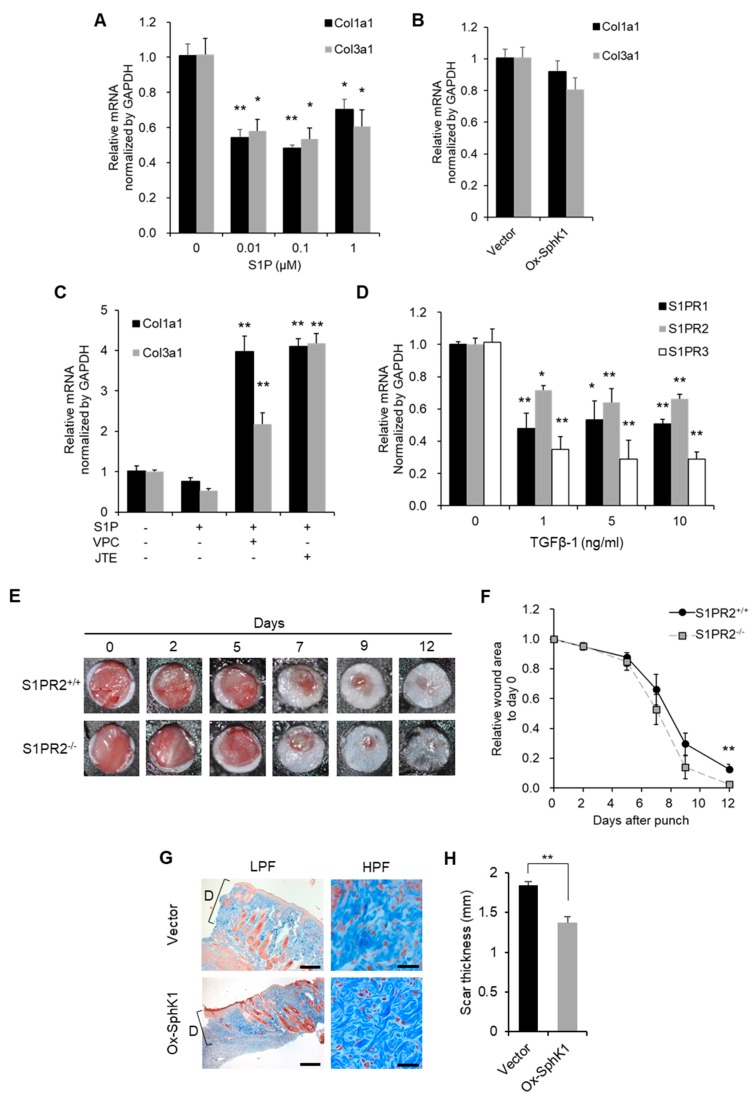
Topical SphK1 gene delivery inhibits scarring. The mRNA expression of Collagen1a1 and Collagen3a1 in NIH3T3 cells (**A**) stimulated with the indicated concentration of S1P for 24 h (*n* = 4) or (**B**) transfected with SphK1-expressing plasmid or vector plasmid (*n* = 4). (**C**) The mRNA expression of Collagen1a1 and Collagen3a1 in NIH3T3 cells stimulated with 1 µM S1P for 24 h with or without 10 µM VPC23019 (inhibitor of S1PR1 and S1PR3) or JTE013 (inhibitor of S1PR2) (*n* = 3). (**D**) The mRNA expression of the indicated S1PRs in NIH3T3 cells stimulated with the indicated concentration of transforming growth factor (TGF)-β1 for 18 h (*n* = 3). (**E**) Splinted excisional wounds were generated in S1PR2^−/−^ and S1PR2^+/+^ mice. Representative images of the closing wounds are shown. (**F**) The wound area over time was measured (*n* = 6). (**G**) Representative images of Masson’s trichrome-stained scars at the point of epithelization after treatment with sCA-encapsulated plasmid ointment (the scale bars are LPF: 400 µm; HPF: 50 µm). “D” indicates the scar thickness. (**H**) The scar thickness was measured and graphed (*n* = 4–6). All values in this figure represent the mean ± s.e.m. * *p* < 0.05, ** *p* < 0.01.

We treated the dermal fibroblast line NIH3T3 with SphK1 plasmid or exogenous S1P. We found that exogenous S1P, but not the SphK1 plasmid, suppressed the transcription of Collagen1a1 and Collagen3a1 in the cells (Figure 7A,B). Thus, exogenous S1P, but not endogenously produced S1P, prevents the collagen deposition of dermal fibroblasts. Notably, when the exogenous S1P-stimulated cells were treated with the S1PR1 and S1PR3 inhibitor VPC23019 or the S1PR2 inhibitor JTE013, their collagen production was restored (Figure 7C). Thus, exogenous S1P suppresses the collagen deposition of dermal fibroblasts via S1PR signaling. Transforming growth factor (TGF)-β1, which is produced during the proliferative phase of wound healing, induces fibroblasts to produce granulation tissue in vivo and extracellular matrix in vitro [33,34]. Our finding that endogenous S1P, but not exogenous S1P, also promotes granulation and participates in the proliferative phase of wound healing led us to examine the effect of TGF-β1 treatment on S1PR expression by NIH3T3 cells. We found that this treatment significantly suppressed transcription of S1PRs (Figure 7D). This suggests that TGF-β1 is a key modulator of the ability of S1P to promote the proliferative phase of wound healing. Given that S1PR2 expression in the wound increased around the end of wound closure (Figure 1C), S1PR2^−/−^ mice had significantly smaller wounds on Day 12 after injury (Figure 7E,F). This suggests that S1PR2 signaling negatively regulates SphK1-S1PR1 signaling, thereby slowing down wound closure at the end of the proliferative phase and allowing the wound to prepare for the remodeling phase. Interestingly, we discovered that when the wounds in the mouse excisional wound splinting model were treated with SphK1-sCA ointment, the scars that formed when epithelization was completed were much thinner than the scars of the vector-sCA-treated mice. The SphK1-sCA-treated wounds also had much thinner collagen bundles, as shown by high power field images (Figure 7G,H).

## 3. Discussion

The present study demonstrates that there were high levels of SphK1 expression after wounding and that high endogenous production of S1P via SphK1 plasmid transfection accelerated wound closure and induced less scarring. It also suggested that the scarless healing induced by the SphK1 plasmid is due to the anti-fibrotic effect of S1P-S1PR signaling in the early phase of wound healing. This is supported by the fact that S1P treatment of dermal fibroblasts suppressed their production of collagens and that this effect was reversed by S1PR inhibitors. These observations together suggest that since the SphK1 plasmid transfection increases S1P levels, it may prolong the anti-fibrotic effects of S1P-S1PR signaling during the proliferative phase of wound healing. This in turn limits the deposition of extracellular matrix, thus generating minimal granulation tissue and very thin scars when epithelialization is complete.

We found that SphK1 knockout significantly decreased the recruitment to the wound of CD3a^+^ T cells but not CD4^+^CD3a^+^ and CD8a^+^CD3a^+^ T cells. By contrast, topical overexpression of SphK1 significantly increased the CD4^+^CD3a^+^ and CD8a^+^CD3a^+^ T cell populations as well as the total CD3a^+^ T cell population in the wound. This apparent discrepancy may reflect the different methods involved: SphK1 knockout has systemic effects whereas the SphK1 ointment has direct local effects. It will be of interest to further examine the profile of the T cells that are recruited to the wound (including NK cells and γδ T cells) when SphK1 is systemically knocked out: this may help elucidate the mechanisms by which the SphK1/S1P axis recruits T cells. Our finding that altering SphK1/S1P signaling shapes not just wound healing but also T cell recruitment suggests that T cells participate in wound healing. This notion is supported by multiple lines of evidence. For example, CD3 T cell numbers in the wound bed increase during the proliferation phase, particularly in the regenerating epidermis [35], and total T cell depletion impairs wound healing [36]. Moreover, burn injuries activate γδ T cells at the injury site. This initiates extensive infiltration by αβ T cells, which facilitate the transition from the inflammatory phase to the proliferative phase [37]. In addition, wound healing is associated with activation of epidermal T cells and their production of growth factors [38].

Overexpression of SphK1 increases recruitment of lymphocytes and macrophages while at the same time enhancing retention; this strengthens subsequent fibroblast activation. Therefore, granulation is promoted. However, fibroblasts activated by TGFβ-1 find it difficult to respond to S1P stimulation as S1PRs are down-regulated (Figure 7D). This phenomenon should decrease as wound healing proceeds towards epithelialization. The anti-fibrotic effect mediated by S1PR signaling occurs with epithelialization. Treatment with the SphK1 plasmid strengthens the dynamic transition from the proliferative phase to the remodeling phase, resulting in inhibited scarring (Figure 7F).

TGF-β1 is likely to play a key role in S1P-S1PR1 signaling-induced less scarring. This cytokine is produced during the proliferative phase of wound healing and induces granulation [33,34]. Fibroblasts activated by TGFβ-1 find it difficult to respond to S1P stimulation as S1PRs are down-regulated (Figure 7A,D). This phenomenon should decrease as wound healing proceeds towards epithelialization. The anti-fibrotic effect mediated by S1PR signaling occurs with epithelialization. Treatment with the SphK1 plasmid strengthens the dynamic transition from the proliferative phase to the remodeling phase, resulting in inhibited scarring (Figure 7F). The expression of TGF-β1 during the proliferative phase of wound healing postpones S1PR2 expression in the wound. Thus, when high S1P levels are generated by SphK1 plasmid transfection, the S1PR2 that is eventually produced is less able to suppress anti-fibrotic S1P-S1PR1 signaling. This limits the production of extracellular matrix and thereby inhibits excessive scar formation.

Immune responses play important roles in skin wound healing [39], and the present study suggests that increased expression of SphK1 also improves wound healing by strengthening these responses. These responses result in the production of multiple wound healing factors; they also have an anti-bacterial effect [40,41]. Since all currently available wound healing agents either target angiogenesis or fibroblast function [42,43,44], SphK1 plasmid treatment constitutes a novel approach to wound healing. Moreover, SphK1 plasmid treatment is simple. By contrast, other wound healing agents must be applied in a stepwise fashion: first, infections or necrotic tissues must be removed, followed by treatment with agents that support fibroblast function and/or angiogenesis. Notably, macrophage polarization was suggested recently to participate in wound healing [45,46]. Further studies exploring the effect of the systemic or local SphK1/S1P axis on macrophage polarization may expand the potential usefulness of these molecules in wound management.

In summary, we found that the SphK1/S1P axis accelerated wound healing by increasing angiogenesis and the recruitment of T cells and macrophages, which secreted various wound-related factors. It was also involved in inhibiting excessive scarring because it promoted the anti-fibrotic effect of S1P signaling. This finding suggests that the SphK1/S1P axis may be a novel therapeutic agent that could help limit scarring after surgery and trauma and aid chronic wound management. However, studies that further elucidate the role of the SphK1/S1P axis in wound healing are needed to determine its full clinical potential.

## 4. Material and Methods

### 4.1. Animal Models and Wound Area Analysis

C57BL/6J mice were purchased from Jackson Laboratory. SphK1 KO and S1PR2 KO mice were from Dr. Richard Proia (The National Institute of Diabetes and Digestive and Kidney Diseases (NIDDK) of National Institutes of Health (NIH)). All animal procedures were approved by the Institutional Animal Care and Use Committee of the Virginia Commonwealth University on 1 July 2015 (AD20100) and the Animal Experimental Ethical Review Committee of Nippon Medical School on 7 September 2016 (28-054). The murine excisional wound splinting model was generated as described previously [25]. Mice were anesthetized with isoflurane, and their dorsal hair was removed. Two 5 mm-diameter full-thickness skin punches were generated symmetrically on either side of the midline. Circular 12 mm-diameter silicon rubber splints were punched in the center to generate 6 mm-diameter holes. They were then fixed with instant-bonding adhesive and sutures around the punch wounds. After applying the required ointment, the wounds were dressed with Tegaderm (3M, Maplewood, MN, USA). The wounds were photographed at the indicated time points. The digital photos were analyzed using GIMP 2.8 software. The pixels of the wound area were normalized to the pixels of the inside of the silicon splint. The wound areas at the indicated time points were expressed as ratios relative to the wound area immediately after wounding.

### 4.2. Cells and S1P Preparation

Murine dermal fibroblast NIH3T3 cells, human cervix epithelioid carcinoma HeLa cells, and human embryo kidney HEK293 were cultured in Dulbecco’s modified eagle medium (DMEM) containing 10% fetal bovine serum (FBS). To analyze collagen production, ascorbic acid 2-phosphate (Sigma-Aldrich, Carlsbad, CA, USA) was added to the culture medium to a concentration of 0.2 mM. S1P was purchased from Sigma-Aldrich and 1 mM was sonicated in 4% bovine serum albumin (BSA). Recombinant human TGFβ-1 was purchased from R&D systems (Minneapolis, MN, USA). VPC23019 was purchased from Avanti (Alabaster, AL, USA). JTE013 was purchased from Cayman Chemical (Ann Arbor, MI, USA).

### 4.3. Plasmid Construction, In Vitro Transfection with sCA-Encapsulated Plasmids, and Preparation of Plasmid-sCA Ointment

The murine SphK1 gene was amplified using TaKaRa Ex Taq Hot Start Version (TaKaRa, Kusatsu, Japan). The SphK1-expressing plasmid was then constructed using the pcDNA3.1/V5-His TOPO TA Expression Kit (Invitrogen, Carlsbad, CA, USA). sCA-encapsulated plasmids were prepared as described previously [27,47]. Thus, 4 μL of 1 M CaCl_2_ was incubated at 37 °C for 30 min with 2 μg of plasmid DNA in 1 mL of an inorganic solution (NaHCO_3_, 44 mM; NaH_2_PO_4_, 0.9 mM; CaCl_2_, 1.8 mM; pH 7.5) and then centrifuged at 12,000 rpm for 3 min. After the pellet was dissolved with DMEM, the solution was sonicated in a water bath for 10 min, thus generating sCA-encapsulated plasmids. Cells cultured in 6-well plates for 24 h were incubated with the plasmid-sCA-DMEM solution for 6 h. The medium was then replaced with DMEM containing 10% FBS. After another 48 h, the cells were collected for total RNA isolation. The plasmid-sCA ointment was generated by dissolving the sCA pellet with 50 µL of PBS, mixing it with 100 µg of plasmid DNA, and then mixing the solution into 200 µL of Aquaphor^®^. The four wounds of two mice were each treated once with 250 µL of ointment.

### 4.4. Sponge Granulomas in Mice and Their Injection with Plasmid-sCA

Sponge granulomas were generated in two mice as described previously [32]. Thus, polyvinyl alcohol (PVA) sponges were processed to generate 10 mm-diameter 3 mm-thick sponges. The sponges were irradiated with ultraviolet light and infiltrated with saline for 48 h. The sponges were then transplanted (two per mouse) in the subcutaneous dorsal area in a symmetrical fashion. sCA was mixed with 50 µg of plasmid DNA and pelleted. The pellet was then dissolved in 100 µL of saline containing 0.5% mouse serum albumin. The two sponges on one mouse were injected every other day with 50 µL of sCA-plasmid. The two sponges on the other mouse were injected with sCA-vector. The samples were harvested 14 days after transplantation and subjected to histological analysis. The percentage of total area that was occupied by eosin-positive area in the sponges were analyzed by using ImageJ. Four fields per sponge were analyzed and the averages were calculated.

### 4.5. Flow Cytometry

Cells were isolated from the wound tissues as described previously [6]. Thus, the tissues were cut into small pieces and digested at 37 °C for 90 min in DMEM containing 10% FBS, 1.2 mg/mL hyaluronidase (Sigma-Aldrich), 2 mg/mL collagenase (Sigma-Aldrich), and 0.2 mg/mL DNase I (Sigma-Aldrich). The cell pellets were resuspended in PBS containing 2% FBS and then incubated with anti-CD16/32 antibody (BioLegend, San Diego, CA, USA) for 5 min to block the Fcγ receptors. To measure inflammatory cell recruitment, the wound cell preparations were stained with phycoerythrin (PE)-conjugated anti-Gr-1, allophycocyanin (APC)-conjugated anti-CD3a, PE/CY7-conjugated anti-CD4, or fluorescein isothiocyanate (FITC)-conjugated anti-CD8a antibody (CiteAb, Bath, UK) at 4 °C for 20 min. The lymphocytes were analyzed with FACSDiva (BD, San Jose, CA, USA).

### 4.6. Quantitative RT-PCR

Total RNA was extracted using TRIzol^®^ Regent (Invitrogen). cDNA was synthesized using High Capacity cDNA Reverse Transcription Kits (Applied Biosystems, Foster City, CA, USA). qRT-PCR was performed by using a CFX96 Real-Time System (Bio-Rad, Hercules, CA, USA) with PowerUp SYBR Green master mix (Bio-Rad). GAPDH served as the internal control. The primer pairs used are shown in Table 1. Relative expression was calculated using the 2^−ΔΔ*C*t^ method with correction for different amplification efficiencies.

### 4.7. Immunohistochemistry, Western Blot Analysis, and Scar Thickness Analysis

Paraffin-embedded sections were stained with H&E or Masson’s trichrome stain and primary antibodies against F4/80, Ki67, and CD34. The immunostained sections were developed with VECTASTAIN Universal Elite ABC Kit (Vector, Burlingame, CA, USA). All antibodies were from Abcam (Cambridge, UK). The sections were analyzed using ImageJ. The excised wound tissue was homogenized with liquid nitrogen. Total protein was isolated using 1% NP-40. Equal amounts of protein were separated by SDS-PAGE and then transferred to a nitrocellulose membrane. The membranes were incubated with primary antibodies against V5 (Invitrogen), VEGF, FGF-2 (Santa Cruz Biotechnology, Dallas, TX, USA), IGF-1 (Abcam), or GAPDH (Cell Signaling Technology, Danvers, MA, USA), followed by horseradish peroxidase-conjugated IgG against mouse, rabbit, or goat antibodies (Jackson ImmunoResearch, West Grove, PA, USA). The membranes were developed by using SuperSignal Chemiluminescent Substrates (Thermo Fisher Scientific, Cambridge, MA, USA). The antibodies used are summarized in Table 2. The quantification was performed by using ImageJ. Scar thickness was measured by photographing the sectioned tissues after Masson’s trichrome staining and then using ImageJ.

### 4.8. Statistical Analysis

Wound groups were compared using two-factor repeated measures ANOVA. If three or more wound groups were compared, ANOVA was followed by post-hoc Tukey’s test. Two wound groups were compared using Student’s *t*-test or Welch’s *t*-test after F test. *p* < 0.05 was considered significant. All statistical analyses were performed using Statcel2 software (OMS, Saitama, Japan).

## Figures and Tables

**Table 1:** The primers used for quantitative real-time RT-PCR.

Primer	Bio-Rad Assay ID	Forward (5′->3′)	Reverse (5′->3′)
Col1a1		CGATGGATTCCCGTTCGAGTA	CATTAGGCGCAGGAAGGTCA
Col3a1		GAAGTCTCTGAAGCTGATGGG	TTGCCTTGCGTGTTTGATATTC
GAPDH	qMmuCEP0039581		
S1PR1	qMmuCID0020925		
S1PR2	qMmuCED0004722		
S1PR3	qMmuCIP0028162		
SphK1	qMmuCED0040475		
SphK2	qMmuCED0039969		

**Table 2:** The primary antibodies used for this study.

Antigen	Company	Application	Concentration
F4/80	Abcam	Immunohistochemistry	1:100
Ki67	Abcam	Immunohistochemistry	1:100
CD34	Abcam	Immunohistochemistry	1:100
V5	Invitrogen	Western blot	1:5000
VEGF	Santa Cruz	Western blot	1:200
FGF-2	Santa Cruz	Western blot	1:200
IGF-1	Abcam	Western blot	1:500
GAPDH	Cell Signaling Technology	Western blot	1:1000

## Abbreviations

S1P	Sphingosine-1-phosphate
S1PRs	Sphingosine-1-phosphate receptors
SphKs	Sphingosine kinases
TGF-β1	Transforming growth factor-β1
